# The Cultivation of Arabidopsis for Experimental Research Using Commercially Available Peat-Based and Peat-Free Growing Media

**DOI:** 10.1371/journal.pone.0153625

**Published:** 2016-04-18

**Authors:** Tiffany Drake, Mia Keating, Rebecca Summers, Aline Yochikawa, Tom Pitman, Antony N. Dodd

**Affiliations:** 1 School of Biological Sciences, University of Bristol, Bristol Life Sciences Building, 24 Tyndall Avenue, Bristol BS8 1TQ, United Kingdom; 2 Cabot Institute, University of Bristol, Bristol BS8 1UJ, United Kingdom; University of Tsukuba, JAPAN

## Abstract

Experimental research involving *Arabidopsis thaliana* often involves the quantification of phenotypic traits during cultivation on compost or other growing media. Many commercially-available growing media contain peat, but peat extraction is not sustainable due to its very slow rate of formation. Moreover, peat extraction reduces peatland biodiversity and releases stored carbon and methane into the atmosphere. Here, we compared the experimental performance of Arabidopsis on peat-based and several types of commercially-available peat-free growing media (variously formed from coir, composted bark, wood-fibre, and domestic compost), to provide guidance for reducing peat use in plant sciences research with Arabidopsis. Arabidopsis biomass accumulation and seed yield were reduced by cultivation on several types of peat-free growing media. Arabidopsis performed extremely poorly on coir alone, presumably because this medium was completely nitrate-free. Some peat-free growing media were more susceptible to fungal contamination. We found that autoclaving of control (peat-based) growing media had no effect upon any physiological parameters that we examined, compared with non-autoclaved control growing media, under our experimental conditions. Overall, we conclude that Arabidopsis performs best when cultivated on peat-based growing media because seed yield was almost always reduced when peat-free media were used. This may be because standard laboratory protocols and growth conditions for Arabidopsis are optimized for peat-based media. However, during the vegetative growth phase several phenotypic traits were comparable between plants cultivated on peat-based and some peat-free media, suggesting that under certain circumstances peat-free media can be suitable for phenotypic analysis of Arabidopsis.

## Introduction

Peat extraction can be unsustainable and cause environmental damage, but some peat-free growing media are anecdotally poor for the cultivation of *Arabidopsis thaliana* (Arabidopsis) for experimental purposes. Plant sciences research involving Arabidopsis requires reproducible plant cultivation; for example during developmental or physiological analysis. Here, we compared systematically several experimentally-relevant phenotypic traits of wild type Arabidopsis that was cultivated using a selection of peat-free growing media.

Peat is an organic material formed from the accumulation of dead plant tissue where decomposer activity is very low. Peat often occurs in anaerobic, waterlogged environments [1]. Peat extraction can be damaging to habitats, unsustainable and may contribute to anthropogenic climate change. Peatlands represent a major habitat, comprising over 400 million hectares of land on Earth [2]. However, most natural bog in Great Britain has been destroyed for a variety of reasons, such as extraction of horticultural peat and electricity generation [3]. For example, 6,949 ha of peatland (48%) in UK Special Areas of Conservation are considered to be degraded [4]. It was estimated that in the United Kingdom alone, 2.69 x 10^6^ m^3^ of peat is extracted annually for commercial horticultural and domestic (gardening) purposes, 99% of which is used to produce growing media [4]. The quantity of peat that is used for plant sciences research involving Arabidopsis is not known. Peat extraction has impacted peatland biodiversity, including population declines in the large heath butterfly [5] and reduced genetic variation in native plants [6]. Additionally, archaeological and paleontological sites have been destroyed by peat extraction [7, 8].

Estimates of the UK peat stores vary from 1.5 million hectares to 5 million hectares [9]. The rate of peat accumulation depends on environmental conditions, but is estimated to be slow (around 100 cm every 1000–2000 years [2, 7]). Mechanised milling can remove the entire peat body, making recovery especially slow [3, 10]. The European Commission does not regard peat as a renewable resource because it can be extracted faster than it is formed [4]. European Union legislation previously required 90% of growing media sold by 2010 to be peat-free and the UK government aims to end the use of peat in horticulture by 2030 [11].

Peatlands are highly concentrated organic carbon stores because they are formed from plant material. The average rate of carbon sequestration by peat is reported to be 17.2 g m^-2^ yr^-1^ [12] with peatlands of Great Britain estimated to contain 5.1 billion tonnes of carbon [13]. Therefore, peat formation and extraction can impact the atmospheric concentration of carbon dioxide. It is estimated that in the last 10,000 years, peatland has stored enough carbon to reduce global temperatures by 1.5–2.8°C [2]. Undisturbed peatlands generally capture carbon [9, 13, 14] and disturbing peatland can release carbon and methane into the atmosphere [13].

There are several horticultural alternatives to peat. Coir is a waste product derived from coconut husks, and can have similar or superior performance to peat-based media [15]. Composted organic waste material can be used, but often has high pH and mineral content that requires mixing with peat to make it suitable for horticulture [16–18]. Wood fibre and composted bark are also used as alternatives to peat [19, 20].

Arabidopsis is a core experimental model for plant sciences research due to its small size, compact genome, lack of repetitive DNA, fast generation time, self-fertilisation, high fecundity and ease of transformation [21, 22]. This is underpinned by extensive germplasm resources and commercially-available materials for genomic analysis. Given that much of plant sciences research aims to understand the relationship between genotype or epigenotype and phenotype, the rapid, reliable and consistent growth of Arabidopsis under controlled conditions is crucial for the effective prosecution of research. For example, investigation of the regulatory networks underlying the photoperiodic control of flowering time has frequently involved the reproducible quantification of time and developmental stage of inflorescence emergence under controlled conditions [23]. Another example is the demonstration that plant circadian clocks increase growth, which involved the monitoring of vegetative growth of plants under controlled conditions [24]. In both cases, reproducible plant cultivation was crucial because inconsistent growth may mask information within noisy data, or require substantially larger replicate numbers for an effective hypothesis test. Investigation of gene function in Arabidopsis often involves generation of transgenic lines using Agrobacterium-mediated transformation. The initial transformation rate is low and recovery of the small number of first-generation transformants requires reliable growing media, since seedling loss wastes research resources.

Given that reproducible and reliable plant cultivation is crucial for research involving Arabidopsis, that peat extraction may cause long-term environmental damage, and there is pressure for Universities to reduce their environmental footprint (e.g. UK National Union of Students’ Green Impact Programme), we investigated the performance of Arabidopsis that was cultivated on several types of commercially-available peat-free growing media that are available in the UK, under simulated experimental conditions. We compared experimentally-relevant phenotypes of Arabidopsis cultivated on peat-based and peat-free growing media that reflect some popular peat-free products available in the UK (coir, wood-fibre, composted bark and domestic compost). We anticipate that our findings may provide guidance concerning compost selection and handling for Arabidopsis researchers and research facilities that conduct large scale cultivation of Arabidopsis.

## Materials and Methods

### Plant Material and Growth Conditions

Col-0 and Landsberg *erecta* (L. *er*) seeds of *Arabidopsis thaliana* were cultivated for 14 days under sterile conditions on half-strength Murashige & Skoog media (0.5 MS) dissolved in 1% (w/v) agar, before transfer to growing media. Briefly, seeds were surface sterilized and stratified as described elsewhere [25], then germinated and cultivated at 19°C with 12 h photoperiod under cool white light (photosynthetic photon flux density 100 umol m^-2^ s^-1^).

To investigate growth on peat-free media, 24-compartment horticultural trays were filled with consistent quantities of growing media, supplemented initially with 500 ml of water per tray containing an insecticide (Intercept, Bayer Cropscience). Plants were not supplied with supplemental fertilizers after transfer to the growing medium. Seedlings were cultivated in a temperature-controlled glasshouse, which had a maximum daytime temperature of 20.4°C and minimum night-time temperature of 19.1°C during the experiments. High-pressure sodium lights generated a 16 h photoperiod. Tray position was rotated weekly to reduce positional effects.

### Compost selection

Five growing media that are available in the UK were compared (Table 1), specifically Levington F2 Seed and Modular Compost (peat-based control; Scotts Miracle-Gro, Ohio, USA), Melcourt Sylvamix Peat-Free Growing Media (peat-free, composted pine and conifer bark with 3% coir; Melcourt Industries, Tetbury, UK), Westland 100% Peat Free All-Purpose Compost (peat-free, wood fibre-based; Westland Horticulture, Dungannon, UK), and domestic compost (peat-free, generated from composted food waste, garden waste, shredded cardboard and paper in Bristol, UK). We also investigated the performance of Arabidopsis on Melcourt Coir Substrate (peat-free, formed from coir alone; Melcourt Industries, Tetbury, UK), which is designed to be used either alone or mixed with other media. Media were compared as supplied and also after autoclaving, with the exception of the domestic compost which was used only in autoclaved form to prevent pest introduction to glasshouses. Therefore, we compared 9 compost types: Levington F2, Melcourt Coir Substrate, Melcourt Sylvamix and Westland Peat Free in autoclaved and native forms, and domestic compost in autoclaved form only.

**Table 1:** Composition of growing media tested, as provided by manufacturers.

Growing medium	Composition
Levington F2 Seed & Modular Compost	Major constituent: Sphagnum moss peat, also 5–10% dolomite, 1–5% calcium nitrate, 1–5% ammonium sulphate
Melcourt Coir Substrate	Horticultural grade washed and buffered coir
Westland 100% peat-free all-purpose compost	Crushed spruce trees, composted bark, slow-release fertilizer, coir
Melcourt Sylvamix peat-free growing media	97% composted pine and conifer back, 3% coir
Domestic Compost	Variable proportions of composted food waste, garden waste, shredded cardboard and paper

### Physiological and developmental phenotypes

*Seedling establishment*. One week after transferring seedlings to growing media, seedlings were scored as ‘established’ if new leaves were produced. *Visible leaf area*. Visible leaf area was determined for the vegetative (rosette) developmental stage only. Images of plants were taken daily, beginning three days after transfer to growth media. Total visible leaf area was calculated using the ImageJ software (imagej.nih.gov/ij/). When bolting occurred, area measurement ceased for that individual and when all individuals in a treatment bolted, rosette area measurement ceased. *Dry biomass at bolting*. Aerial tissue was removed and dried in an oven (80°C for 3 days) and weighed to determine dry biomass. *Time to bolting*. Plants were scored as bolted when the inflorescence exceeded 1 cm; at this point the number of days between germination and bolting was recorded. *Photosynthetic performance*. Basic photosynthetic parameters were compared using a JUNIOR-PAM modulated chlorophyll fluorometer (Heinz Walz GmbH, Effeltrich, Germany). Plants were dark-adapted for 30 minutes, and Fv/Fm measured from two leaves of every living plant at the time of bolting. Data were acquired and processed using the WinControl-3 software (Walz). *Seed quantity*. Seed yield from each plant was estimated by the total weight of seed produced by the plant, calibrated to the weight of 50 seeds counted individually for each treatment. *Seed viability*. Seeds harvested freshly were surface-sterilised and plated on 0.5 MS as described above. For each of three replicate parent plants, 32 seeds were tested, divided equally across two petri dishes. Seeds were stratified for three days at 4°C, with Petri dishes wrapped in two layers of foil, before transfer to a growth chamber at 19°C with a 12 h photoperiod. Germination was scored 5 days later using a dissection microscope, with germination defined as radicle emergence from the seed coat and endosperm [26].

#### Quantification of properties of growth media

*pH and nutrient composition*. A soil test kit (Professional Soil Test Kit, Testwest, Wales) was used to measure pH and NPK (nitrogen, phosphorous and potassium) for each growth medium. Measurements were conducted on a separate aliquot of compost rather than compost removed from trays containing seedlings. *Water retention*. 4 L of each growth medium and 500 ml of water were combined and placed within 24-compartment disposable horticulture trays, identical to those used for phenotyping, in duplicate for each growth medium. Trays were weighed immediately after adding water then left in glasshouse conditions identical to those used for phenotyping. Trays were weighed at the same time every day for 5 days, then every 3 days subsequently to record water loss. *Media particle size*. 200 g of each growing medium was processed through 5.60 mm and 2.00 mm soil sieves and the amount of material in each size fraction weighed to obtain information concerning growth media texture. *Powdery mildew and algae*. The number of horticultural tray compartments containing powdery mildew and/or algae was determined by scoring a tray compartment as being infected with mildew if any white material was present on the surface of the media, and scored as contaminated with algae if the surface of the compost had a green colouration. In all cases, the parameters were quantified using unadulterated media that had not been used for cultivation and that lacked insecticide.

### Statistical analysis

For all data, homogeneity of variance assumptions were tested using a Levene’s test. If assumptions were met, an ANOVA and Tukey post-hoc test were used. If the assumptions were not met, a Welch’s test and Games-Howell post-hoc test were used, as these are appropriate for groups with unequal variance. Analysis was conducted using SPSS.

## Results

### Seedling establishment

**Fig 1:**
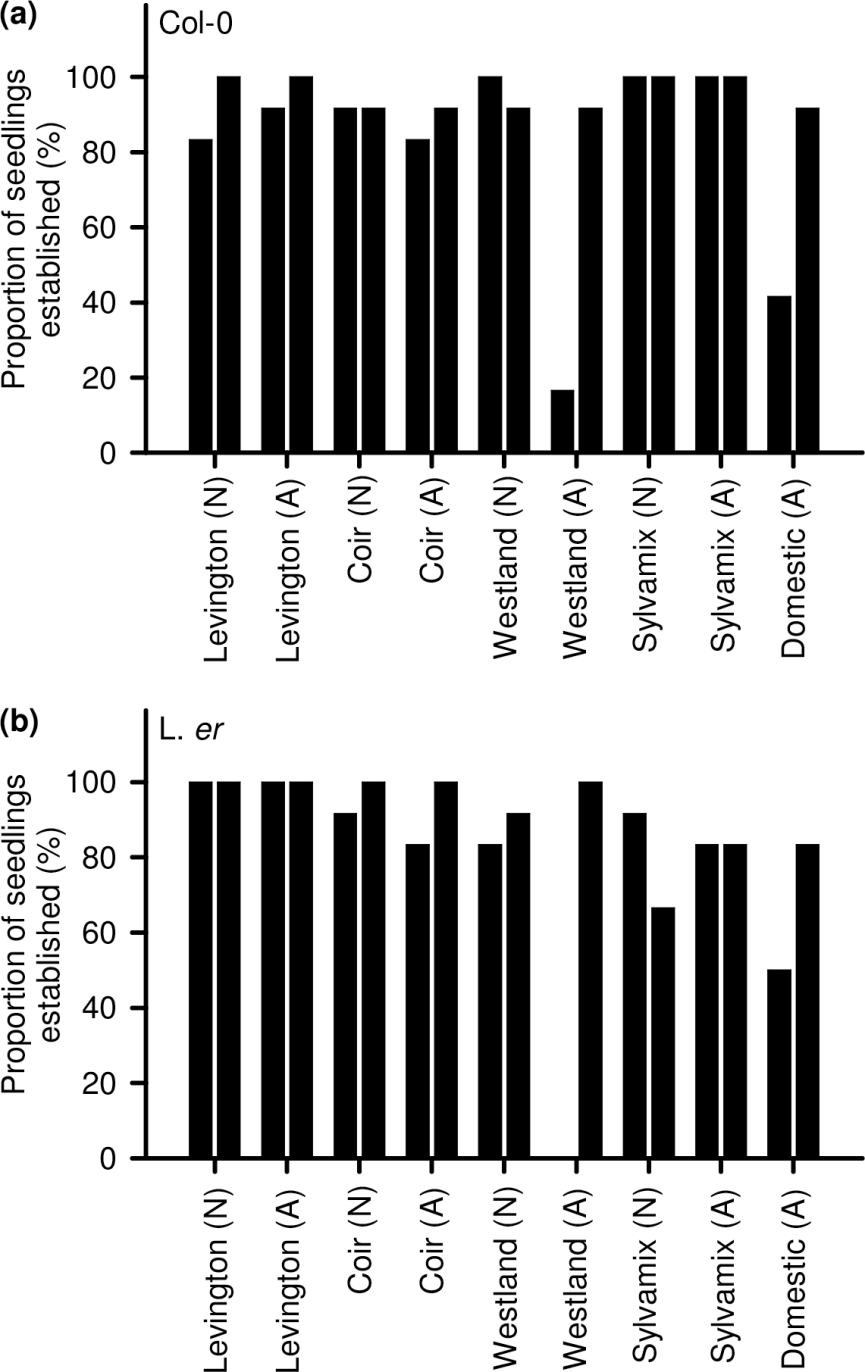
Establishment of Arabidopsis seedlings on peat-based and peat-free growing media. The proportion of seedlings that continued to produce leaves after transfer from sterile culture to growing media was determined in two experimental repeats for (a) Col-0 and (b) L. *er*. backgrounds. The experiment included a comparison of native (N) and autoclaved (A) media. N = 12 per experimental repeat.

A very high proportion of seedlings established successfully on all growing media, following transfer from agar, except for autoclaved Westland media and domestic compost (Fig 1A and 1B). In one experimental repeat, very few seedlings established successfully in autoclaved Westland media due to powdery mildew within the compost, whereas seedlings established well when this media was infection-free. In the absence of infection, there was also variability in seedling establishment when domestic compost was used (Fig 1A and 1B). This could be associated with batch-to-batch heterogeneity of this medium compared with commercial growing media. In Sylvamix, Col-0 seedling establishment was similar to the Levington control medium, but L. *er*. seedlings had reduced establishment frequency in both trials (Fig 1B). For both backgrounds, seedlings established less well in domestic compost, with fewer than half of seedlings surviving in one experimental repeat and less than 100% establishment in the other repeat (Fig 1A and 1B).

### Rosette expansion

**Fig 2:**
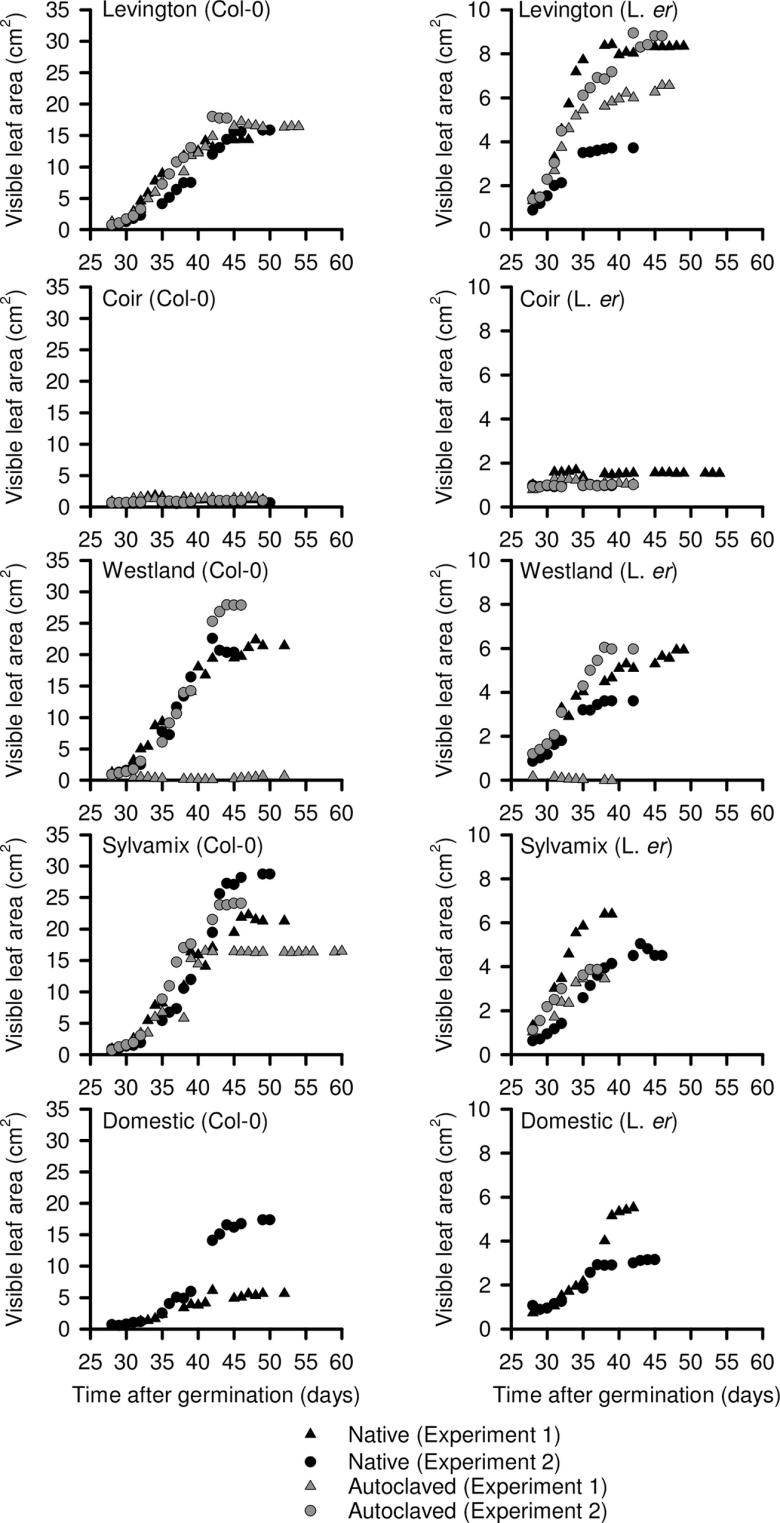
Rate of leaf expansion of Arabidopsis seedlings on peat-based and peat-free growing media. Visible leaf area was determined by image analysis of Col-0 and L. *er*. plants for the period of time between transfer of seedlings to growing media and inflorescence emergence. N = 12 per experimental repeat.

**Fig 3:**
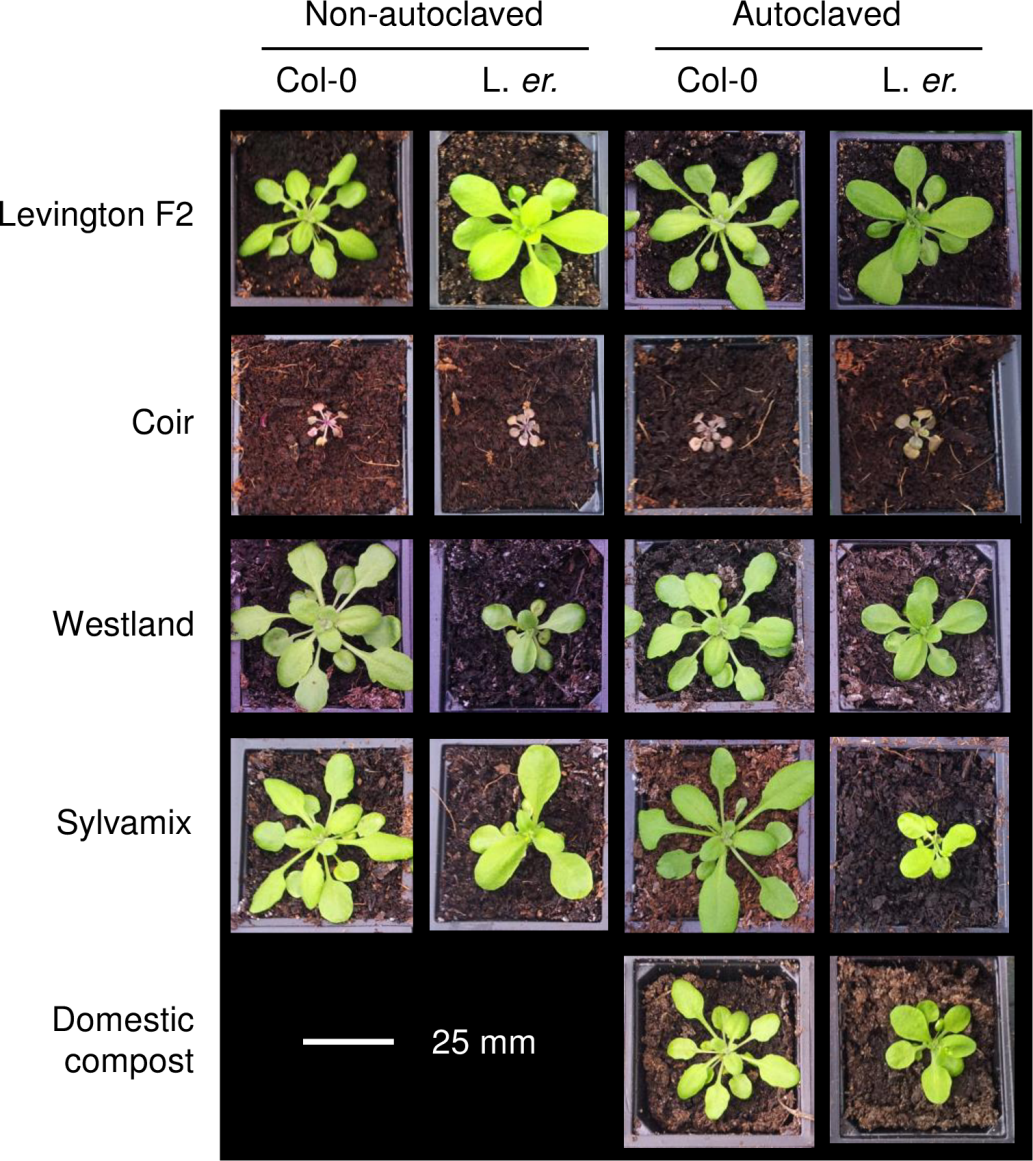
Representative images of Arabidopsis plants cultivated on each growing media. Images are to scale, colours are not calibrated between images, and were taken 11 days after transfer to growing media.

Seedlings cultivated on Levington peat-free control, Westland and Sylvamix media had comparable rates of rosette expansion, with the exception of experimental repeat 1 using autoclaved Westland media, where substantial mildew infection occurred (Fig 2). Rosette expansion on domestic compost was generally lower than when commercial peat- or wood-based media were used (Fig 2). There was no obvious effect of autoclaving Levington peat-based control media upon rosette expansion of Col-0 plants, whereas autoclaving appeared to increase the consistency of growth of L. *er* between trials (Fig 2). Representative images of Arabidopsis plants cultivated on each growing medium are shown in Fig 3.

### Biomass accumulation

**Fig 4:**
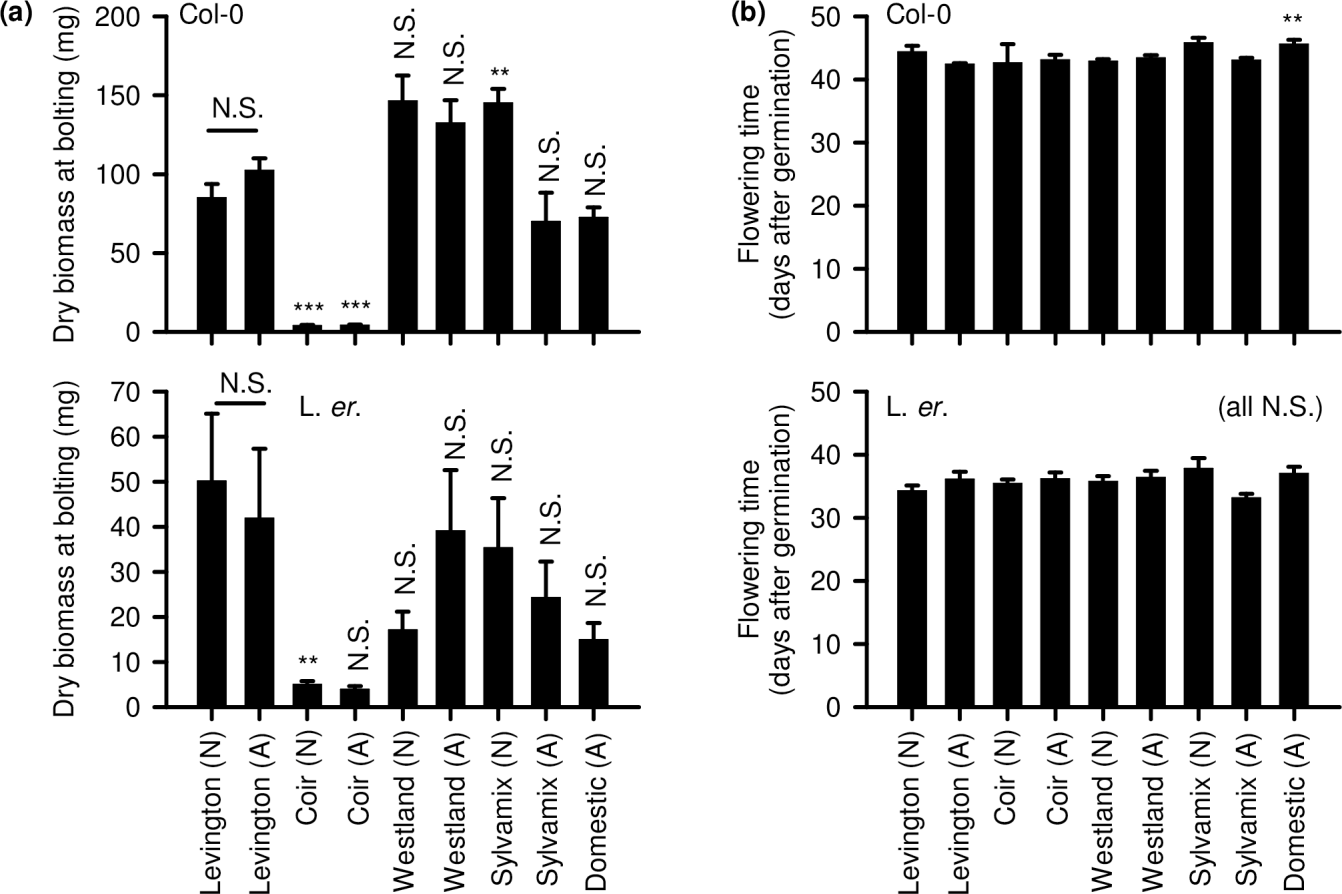
Dry biomass accumulation and time to flowering of Arabidopsis cultivated on peat-based and peat-free growing media. (a) For dry biomass quantification and (b) flowering time, analysis was by Welch’s test (for biomass both Col-0 and L. *er*. P < 0.001; for flowering time Col-0 P < 0.001; L. *er*. P > 0.05 all NS). (a, b) used post-hoc Games-Powell analysis. Significance levels are indicated for pairwise comparisons of peat-based Levington control (native or autoclaved) with other media types that were treated in the same way, and also comparing autoclaved and native Levington media. N = 8–12. The experiment included a comparison of native (N) and autoclaved (A) media.

Growing media choice had a significant impact upon biomass accumulation of both L. *er*. and Col-0 backgrounds (L. *er*. p < 0.001; Col-0 p < 0.001). Seedlings cultivated on coir accumulated very little biomass (Fig 4A), consistent with coir-cultivated seedlings having lowest rates of rosette growth (Fig 2). L. *er*. background seedlings cultivated on non-autoclaved Westland and Sylvamix media, and on autoclaved domestic compost, had numerically lower biomass accumulation compared with the Levington media control, but these differences were not significant due to the degree of biomass variation within each of the treatments (Fig 4A). Col-0 background seedlings cultivated on non-autoclaved Sylvamix media accumulated significantly greater biomass compared with seedlings grown on peat-based Levington media (Fig 4A).

We analyzed variation in biomass accumulation, because consistency of seedling cultivation is important for experimental purposes. L. *er* seedlings cultivated on the Levington peat-based control had the greatest variation in biomass accumulation, so grew less consistently than L. *er*. in peat-free growing media (Fig 4A). In comparison, Col-0 seedlings grew less consistently in wood-based growing media (Westland, Sylvamix) than the peat-based Levington control (Fig 4A; pairwise Levene’s tests revealed non-autoclaved Westland and autoclaved Sylvamix had significantly larger group variance than autoclaved Levington media; p = 0.03 and p = 0.001).

### Flowering time

There were no differences in the time to bolting of Col-0 and L. *er*. plants cultivated on commercially-available peat free growing media compared with the Levington peat-based control (Fig 4B). Col-0 plants cultivated on domestic compost flowered significantly later than Col-0 cultivated on all other growing media (Fig 4B).

### Photosynthetic efficiency

**Fig 5:**
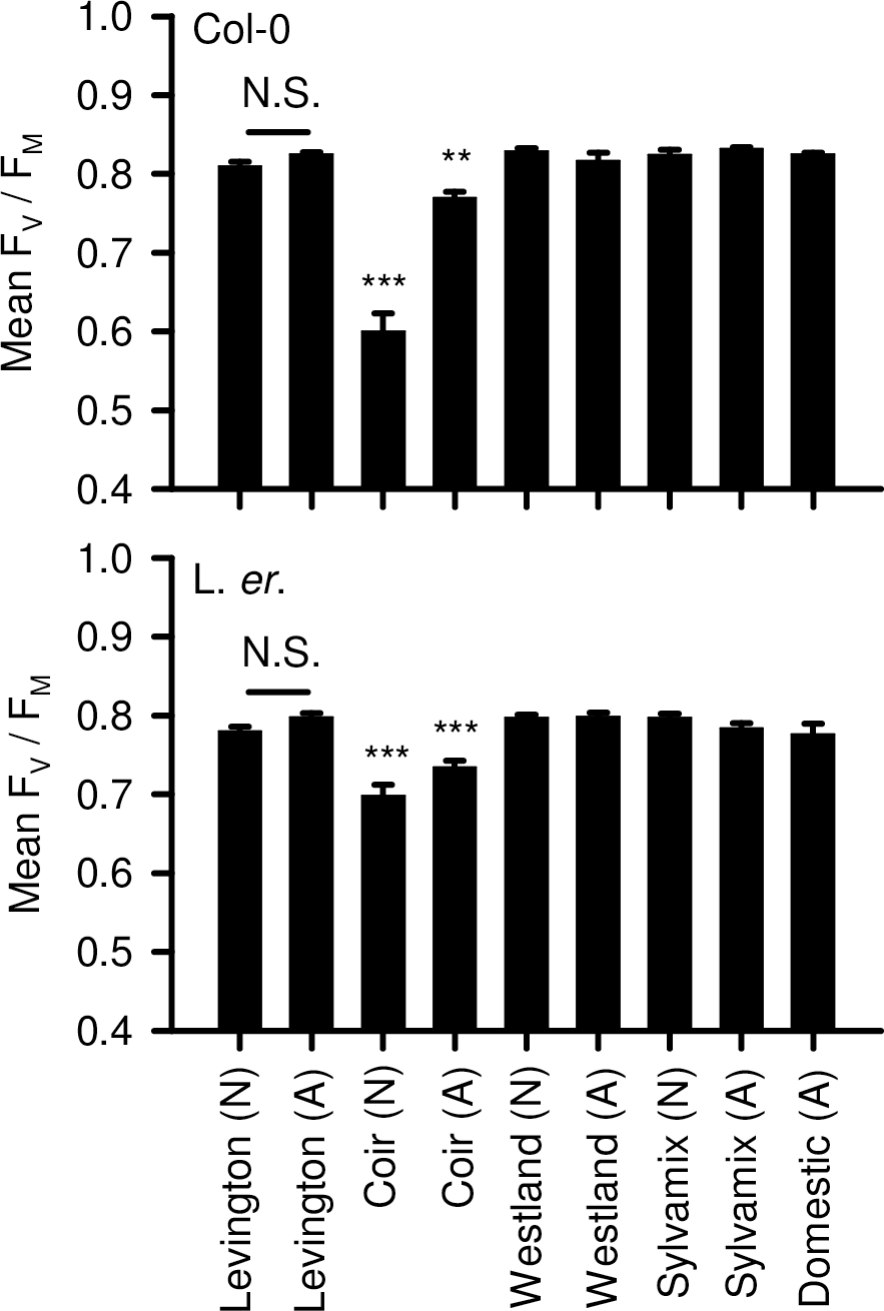
F_V_ / F_M_ measure of maximum photosynthetic yield of PSII of Arabidopsis cultivated on peat-based and peat-free growing media. Significance indicated for pairwise comparisons of Levington peat-based media (native or autoclaved) with other media types (native or autoclaved); Col-0 P < 0.001; L. er. P < 0.001 (Welch’s test followed by post-hoc Games-Howell analysis; N = 12, dead plants excluded). Unmarked comparisons were not statistically significant. The experiment included a comparison of native (N) and autoclaved (A) media.

We used a measure of photosynthetic efficiency to evaluate the degree of abiotic stress arising from cultivation of Arabidopsis upon the growing media that we studied. The ratio of variable chlorophyll fluorescence to maximum chlorophyll fluorescence in dark-adapted plants (Fv/Fm) provides a measure of maximum quantum yield of Photosystem II photochemistry, and decreases in Fv/Fm below a typical maximum value of ~0.83 can indicate damage to the light harvesting apparatus (Maxwell & Johnson 2000). For both Col-0 and L. *er*., cultivation on coir caused a large reduction in Fv/Fm (Fig 5) whereas plants cultivated on other peat-free media had similar Fv/Fm to the Levington control (Fig 5). This suggests there was photoinhibitory damage to PSII in seedlings cultivated on coir. This was consistent with other stress responses during cultivation on coir alone, such as increased anthocyanin accumulation suggested by the purple colouration of leaves (Fig 3).

### Seed yield and viability

**Fig 6:**
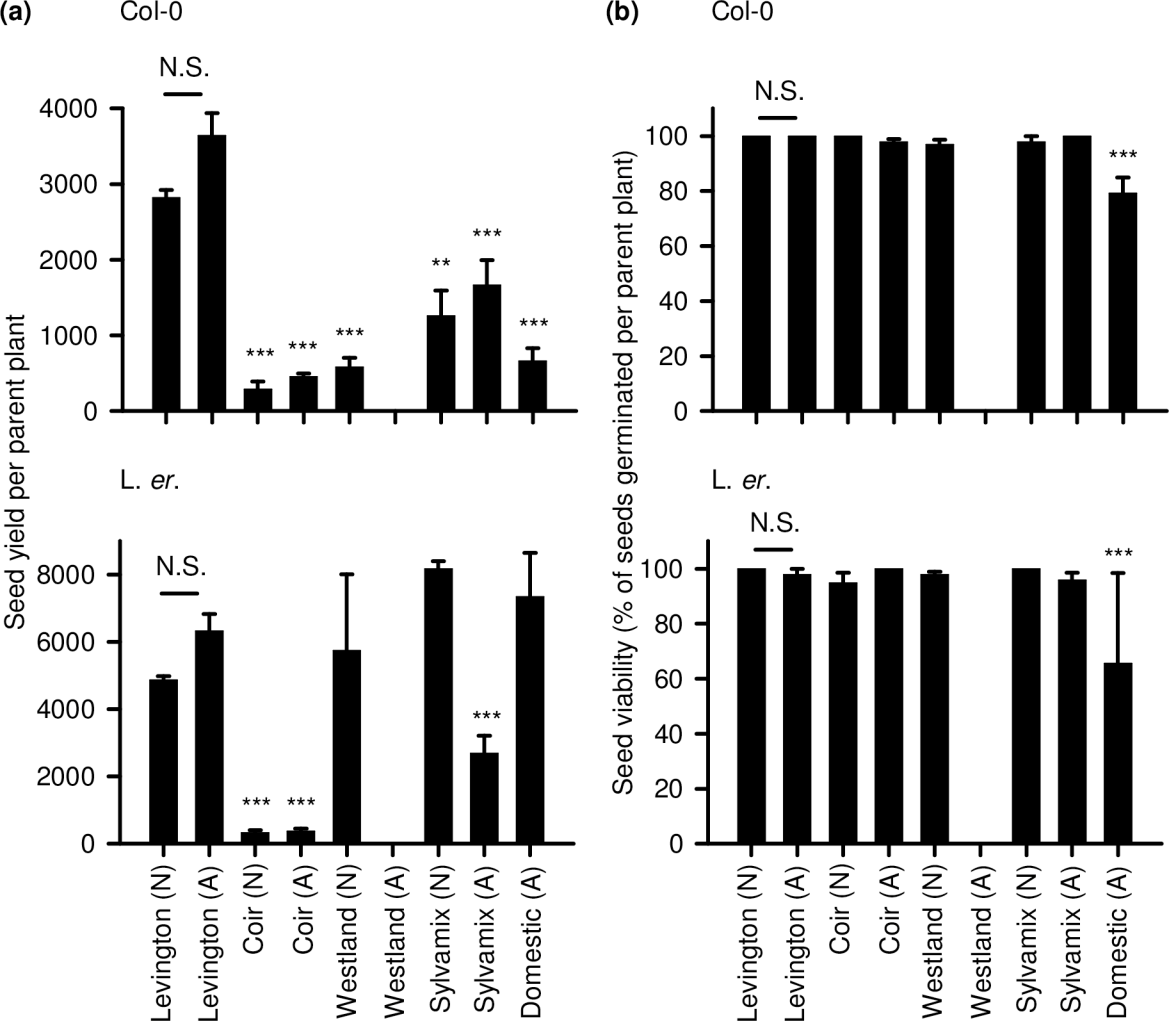
Yield and germination of seed from Arabidopsis cultivated on peat-based and peat-free growing media. (a) Seed yield per plant (N = 3; Col-0 P < 0.001; L. *er*. P < 0.001; Welch’s test followed by post-hoc Games-Howell analyis); (b) Seed viability expressed as the mean proportion of seed germinating from each of 3 parent plants. In some instances, s.e.m. is zero because germination was 100% across seed derived from all parent plants. The experiment included a comparison of native (N) and autoclaved (A) media.

Experimental research with Arabidopsis often requires the reliable generation of large quantities of high quality seed. Therefore, we assessed the quality and quantity of seed that was produced following the cultivation of Arabidopsis on several peat-free growing media. Col-0 plants produced significantly fewer seeds after cultivation on all peat-free media compared with the Levington peat-based control (Fig 6A). L. *er*. plants produced significantly fewer seed when grown on coir (Fig 6A). Interestingly, L. *er*. plants cultivated on wood-based growing media (Westland, Sylvamix) produced quantities of seed that were comparable with the peat-based control when the media was not autoclaved, but seed yield was reduced significantly on autoclaved wood-based growing media for L. *er*. (Fig 6A). No seed was obtained from Col-0 or L. *er*. plants cultivated on autoclaved Westland wood-based media (Fig 6A). Although numerically more seed was obtained from both background lines when cultivated on Levington peat-based media that was autoclaved relative to non-autoclaved Levington media, this was not statistically significant (Fig 6A).

Plants cultivated on all media produced seeds that germinated reasonably well (Fig 6B), although the viability of seed derived from both Col-0 and L. *er*. cultivated on domestic compost was reduced significantly. Since no seed was obtained from plants cultivated on autoclaved Westland media, we cannot provide a measure of viability (Fig 6A and 6B). Autoclaving peat-based control media (Levington) had no effect upon seed viability (Fig 6B).

### Mildew and algal contamination

**Fig 7:**
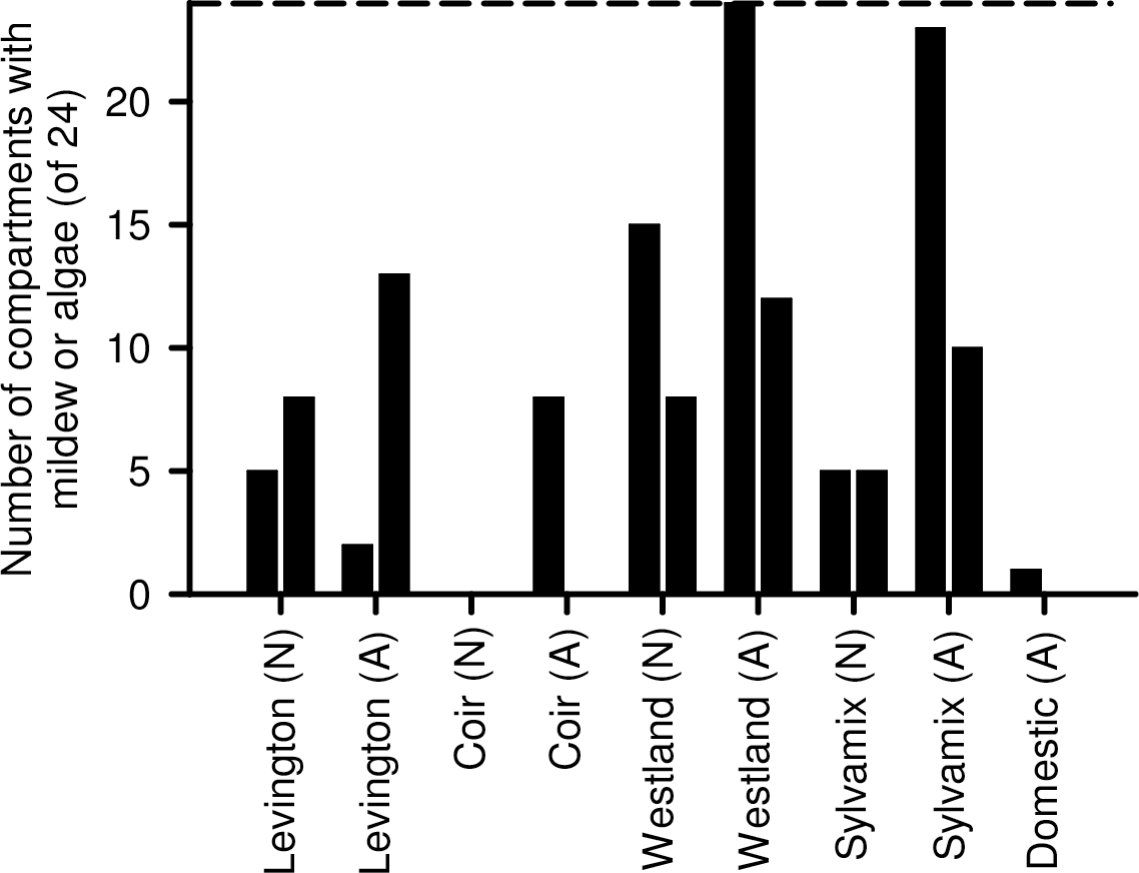
Proportion of compartments of horticultural trays that had mildew or algae on media surface, determined by visual inspection of two independent repeats of each growing medium. The experiment included a comparison of native (N) and autoclaved (A) media. Trays had 24 compartments, with the broken horizontal line indicating this maximum number of compartments with the potential for infection.

The presence of mildew or algae on the surface of the growing media was assessed visually. There was considerable variation in the degree of contamination between experimental repeats (Fig 7). Autoclaving Levington, Westland and Sylvamix media increased the likelihood of contamination of individual compartments on growing trays with mildew or algae.

### Physical and chemical properties of growing media

**Fig 8:**
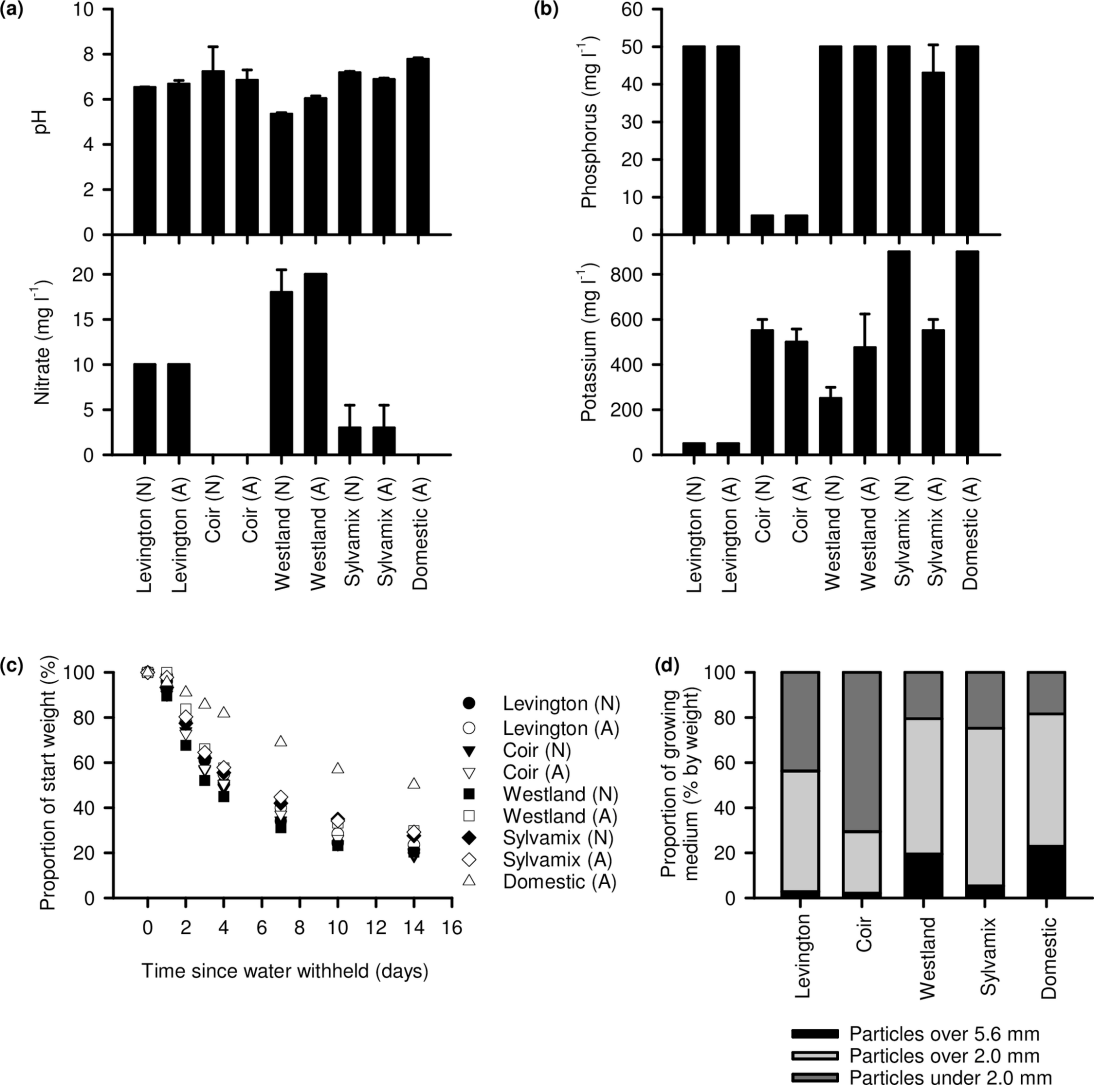
Chemical and physical properties of growing media compared in the study. (a) pH and nitrate composition and (b) phosphorus and potassium content of media (N = 4). (c) Water retention of growing media in the absence of plants, under our growing conditions (determined from two horticultural trays of 24 compartments each per growing media). (d) Particle size distribution of non-autoclaved growing media. (a, b, c) For each growing medium, N indicates native (non autoclaved) and A indicates that the medium was autoclaved.

We compared key physical properties of the growing media (Fig 8). Peat-based Levington media, Sylvamix wood-based media and coir were roughly pH neutral, whereas Westland was slightly acidic, and domestic compost slightly alkaline (Fig 8A). Coir, Sylvamix and domestic compost had low nitrate content compared with the Levington control and Westland media (Fig 8A). Coir was also low in phosphorous and the Levington control contained relatively little potassium compared with other media types (Fig 8B). The growing media lost water to the atmosphere at similar rates, except for domestic compost, which retained water for longer (Fig 8C). The growing media tested had significant differences in the proportion of particles ≤ 2mm (p < 0.001). Coir was dominated by smaller particles than the Levington peat-based control media, whereas Westland wood-based compost and domestic compost contained rather more large particles (p < 0.001 in all cases; ANOVA and post-hoc Tukey analysis). This means that overall, coir had a very fine texture whereas Westland, Sylvamix and Domestic had coarser textures than the Levington peat-based control.

## Discussion

Our data indicate that coir alone is unsuitable for cultivation of Arabidopsis for research purposes, as this media caused poor performance across almost all parameters relative to the peat-based control. This might relate to a lack of mechanical support within the fine texture of coir, or its lack of nitrate (Fig 8A and 8D). It is possible that addition to coir of organic material having suitable nutrients, or watering with a nutrient solution such as MS salts, might improve the experimental cultivation of Arabidopsis on coir media. Although Arabidopsis cultivated on domestic compost did not perform as well as when cultivated on peat-based media, domestic compost might be used as partial substitution for peat within bespoke laboratory compost mixes. In many cases, wood-based growing media performed as well as the peat-based medium for cultivation of Arabidopsis; seedlings established well and grew quickly, accumulating biomass and flowering in an almost identical manner to the peat-based medium. However, plants cultivated on wood-based media had inconsistent seed production (Fig 6A) and were vulnerable to fungal infection, especially after media was autoclaved (Fig 7). Decreased seed yield from plants cultivated on coir and wood-based media, relative to the Levington peat-based media, might arise from reduced self-fertilization or poor seed formation, caused by either reduced photosynthate generation (particularly coir; Fig 5) or differing macronutrient compositions (Fig 8A and 8B). For example, nitrogen availability can regulate the developmental progression of Arabidopsis, including seed development [27, 28]. Reliable generation of large quantities of seed from Arabidopsis, under our experimental conditions, was only possible with peat-based growing media. However, during the vegetative growth phase several phenotypic traits examined were comparable between peat-based and wood-based (peat-free) growing media (Fig 1 Fig 2, Fig 3, Fig 4), suggesting that commercially-available peat-free growing media can be suitable for experimental research involving Arabidopsis.

We are aware that researchers sometimes need to decide whether compost should be autoclaved before conducting experiments. Our data enable the side-by-side comparison of the impact of autoclaving growing media upon the cultivation of Arabidopsis within standard peat-based growing media. There was no overall impact of autoclaving of peat-based media upon the parameters that we measured, although autoclaved media was more frequently infected with mildew or algae (Fig 7). Reasons why autoclaved media might become infected more frequently include (i) killing of native microbiota creates a pristine medium that can become infected rapidly with new microbes in the absence of competition [29, 30], (ii) high temperatures used by autoclaving increase the concentration of soluble organic carbon [29, 30], creating an enriched growth medium, and (iii) high temperatures alter the mobility and chemical state of toxic mineral salts, other cations and ammonium [31, 32], causing alterations in the microbial composition of the medium. Laboratory autoclaves typically heat media to 121°C or greater, whereas commercial soil sterilization is normally conduced at 82°C for > 30 minutes to avoid problems of this nature [33].

From a whole life-cycle perspective, our data indicate that Arabidopsis performs best on peat-based growing media, compared with its performance under identical conditions on several commercially-available peat-free media (Table 1). Nevertheless, during the vegetative growth phase some phenotypic characteristics were comparable between peat-based and certain commercially-available peat-free media. When interpreting data for publication, it is important to be aware that choice of growing medium can affect phenotypic traits, and do so in an ecotype-specific manner, which could underlie variability in experimental reproducibility between laboratories. Our standard laboratory protocols, glasshouse conditions and watering regimes were optimized for working with peat-based growing media. It is possible that the optimization of growth conditions, such as altered watering regimes with peat-free media, supplementation of peat-free media with additional nutrients, or preparation of bespoke peat-free compost formulations, will allow the reduction of peat use in experimental research involving Arabidopsis with no impact upon experimental performance.
